# Nonvitamin K oral anticoagulants with proton pump inhibitor cotherapy ameliorated the risk of upper gastrointestinal bleeding

**DOI:** 10.1038/s41598-023-44494-9

**Published:** 2023-10-13

**Authors:** Parata Chaiyana, Karjpong Techathuvanan, Supatsri Sethasine

**Affiliations:** 1 Department of Medicine, Faculty of Medicine, Vajira Hospital, Navamindradhiraj University, Dusit, Bangkok, 10300 Thailand; 2 Division of Gastroenterology and Hepatology, Department of Medicine, Faculty of Medicine, Vajira Hospital, Navamindradhiraj University, 681 Samsen Road, Dusit, Bangkok, 10300 Thailand

**Keywords:** Cardiology, Gastroenterology

## Abstract

Proton pump inhibitors (PPIs) can reduce the risk of upper gastrointestinal bleeding (UGIB) in patients who are taking oral anticoagulants. This study aimed to identify the association between NOACs with PPI cotherapy and UGIB. This retrospective cohort analysis included patients over the age of 18 years who were using NOACs between 2013 and 2020. NOAC categories, concomitant medications, endoscopic findings, the HAS-BLED score and the Charlson Comorbidity Index score were recorded. Using Poisson regression models, the relationship between UGIB events and risk factors was analyzed. Throughout a mean follow-up of 29.5 months, 14 (5.1%) individuals experienced UGIB. The incidence of UGIB was greater in patients receiving NOACs without PPIs (2.7 [1.26–5.60] per 1000) than in those receiving NOACs with PPIs (1.3 [0.61–2.67] per 1000). Patients receiving NOACs with PPIs had a 79.2% lower incidence of UGIB than patients receiving NOAC monotherapy (RR 0.208, 95% CI 0.061–0.706; *p* = 0.012). Female sex and the HAS-BLED score were associated with UGIB (RR 5.043; 95% CI 1.096–23.20; *p* = 0.038; RR 2.024; 95% CI 1.095–3.743; *p* = 0.024, respectively). Patients receiving NOAC and PPI cotherapy had a lower incidence of UGIB than those receiving NOACs alone, and female sex was a risk factor for UGIB in NOAC-treated patients.

## Introduction

Nonvitamin K oral anticoagulants (NOACs) are innovative agents generated in a cascade by thrombin inhibitors or direct factor Xa inhibitors. Numerous studies have shown that NOACs are effective in both the prevention and treatment of venous thromboembolism^1–6^. NOACs are gaining popularity because of their rapid onset and offset of action, predictable pharmacodynamics that reduce the need for routine therapeutic monitoring, and fewer dietary or drug interactions in comparison to warfarin^7^. The 2018 European Heart Rhythm Association Practical Guide therefore recommends NOACs as a first-line treatment for atrial fibrillation or patients who have previously taken warfarin with a variable international normalized ratio (INR)^8^.

Anticoagulants have a significant detrimental effect on bleeding, especially upper gastrointestinal bleeding (UGIB). NOACs promote gastrointestinal bleeding (GIB) via multiple mechanisms, such as local and/or systemic anticoagulant effects or by inhibiting gastrointestinal mucosal repair^9^. However, additional evidence has suggested that NOACs are superior to vitamin K antagonists in terms of GIB risk reduction, particularly in patients with a history of GIB, but the risk is not eliminated^10,11^. In a previous study^12^, the use of gastroprotective drugs was associated with a lower incidence of GIB in patients receiving dabigatran. Other studies have discovered that proton pump inhibitor (PPI) cotherapy decreases the incidence of UGIB in patients^13^. However, only a subset of high-risk NOAC-treated patients with concurrent antiplatelet medication use or a history of peptic ulcer or previous GI hemorrhage may benefit from gastroprotective therapy^14^. The relationship between PPIs and NOACs has not been investigated in Thailand, and there are no recommendations for preventing UGIB in NOAC-treated patients. This investigation aimed to determine the relationship between NOACs with PPI cotherapy and UGIB.

## Methods

### Study design

**Figure 1:**
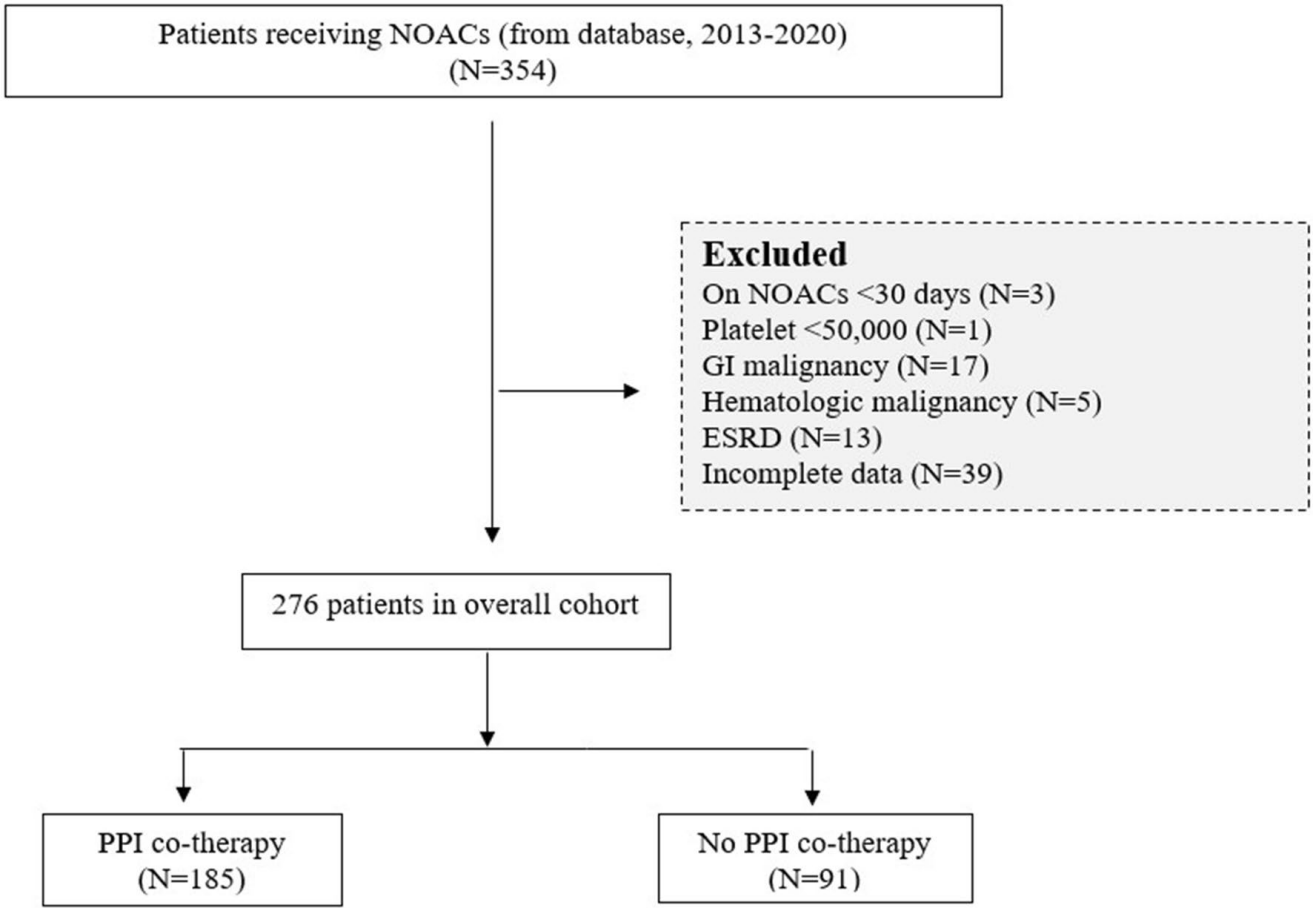
Flow diagram of patients treated with NOACs.

Patients who received NOACs at tertiary centers between January 2013 and December 2020 were included in this retrospective cohort study. Patients were diagnosed with atrial fibrillation or venous thromboembolism at least 90 days before NOACs were initiated. Patients taking NOACs were divided into two groups based on whether they received PPI cotherapy. A flow chart illustrates the investigation methodology (Fig. 1). The investigators examined and extracted data from the Electronic Public Hospital Information System (EPHIS) of Vajira Hospital. Institutional review committees gave approval for the current study. This investigation was approved by the Human Research Ethics Committee of Navamindradhiraj University (COA 207/2564).

### Study population

Eligible patients at the Faculty of Medicine at Vajira Hospital, Navamindradhiraj University, were at least 18 years old and had received NOACs, such as dabigatran, rivaroxaban, apixaban, or edoxaban, continuously for more than 30 days after initial prescription. Eligible patients had comorbidities such as hypertension, diabetes, chronic kidney disease (CKD), cardiovascular disease and cirrhosis. The exclusion criteria included patients with a previous prescription for vitamin K antagonists, less than 30 days of NOAC use, severe thrombocytopenia, gastrointestinal malignancy, hematologic malignancy, or end-stage renal disease (ESRD).

### Exposure and follow-up criterion

We defined patients exposed to NOACs alone as those whose EPHIS data indicated an NOAC prescription for at least 30 days, regardless of the NOAC type. PPI prescriptions were initiated by the clinician based on comorbidities and concurrent drug use. Patients were classified as PPI-exposed if their EPHIS records revealed a minimum daily dose of 20 mg/day for at least 30 days. The PPI-exposed cohort was counted until their last PPI prescription expired. The NOACs-PPI exposure group was defined as patients who took NOACs and PPIs concurrently for at least 30 days. Following PPI and NOAC cotherapy for at least 30 days, patients were classified as PPI-exposed if there was less than 30 days between PPI discontinuation and the onset of UGIB. The standard of care required visits every three months for both groups of patients. Presumed prescription data recorded in the EPHIS regarding good medical adherence was considered to indicate drug compliance.

During the time period documented in this study protocol, patient bleeding events were regularly monitored based on data. The outcome of interest was the diagnosis of UGIB in both inpatient and outpatient settings of the tertiary care departments. We censored patients upon the occurrence of gastrointestinal (GI) hemorrhage, at the conclusion of the study period, if they did not survive, or if their treatment was converted to warfarin therapy.

### Data collection

This study used the EPHIS database. We conducted a retrospective cohort study of patients with atrial fibrillation (AF) or venous thromboembolism using age, sex, and comorbidities as common patient characteristics. Patients receiving antiplatelets, nonsteroidal anti-inflammatory drugs (NSAIDs), and steroids for at least 7 days were evaluated for concomitant use of these medications.

The calculation for the Charlson Comorbidity Index (CCI) was based on the adjusted mortality risk. The score was calculated by adding 1 point for each decade of age over 50 years, up to a maximum of 4 points, and was weighted according to multiple variable comorbidities. The more points that were calculated, the more likely it was that the negative predicted outcome would occur. Scores were interpreted as follows: 0 points = no comorbidity, 1–2 points = mild comorbidity, 3–4 points = moderate comorbidity and > 5 points = severe comorbidity. The HAS-BLED score was calculated to estimate the risk of major hemorrhage in patients receiving NOACs. We collected data on esophagogastroduodenoscopy (EGD) results and hospitalization duration for the UGIB group.

### Endpoint definition

During the time period documented in this study protocol, patient bleeding events were routinely monitored. The outcome of interest was the diagnosis of UGIB according to ICD-10 codes (Supplementary Table S1) in both inpatient and outpatient tertiary care department contexts. We censored patients upon the occurrence of gastrointestinal (GI) hemorrhage, at the conclusion of the study period, if they did not survive, or if their treatment was converted to warfarin therapy.

### Statistical analysis

Based on our pilot study evaluating the incidence of UGIB in patients taking NOACs (7.7%) and NOAC-PPI cotherapy (3.8%), the sample size for each group was 559, calculated by estimating the sample size and power according to the two binomial proportions function of Bernard, R in the Fundamentals of Biostatistics, fifth edition. Continuous data with a normal distribution are displayed as the mean and standard deviation (SD). For data that were not normally distributed, the Mann‒Whitney U test was used. Using the chi-square and Fisher's exact tests, numerical and percentage representations of categorical data were compared. The HAS-BLED score and CCI score are represented by the mean and standard deviation (SD) or the median and interquartile range (IQR). By dividing the number of occurrences by the total duration of follow-up (per 1000 person-months), the incidence rates were calculated.

NOACS users' risk of gastrointestinal hemorrhage can be affected by age, sex, concurrent medication use, clinical comorbidities, the HAS-BLED score, and the CCI score. To reduce confounding factors, the Poisson regression model was used to adjust for age, sex, comorbidities, the NSAID score, antiplatelet use, steroid use, the HAS-BLED score, and the CCI score in the multivariable models. Poisson regression was used to determine the relationship between UGIB and NOAC-PPI cotherapy. Stata version 15.0 of STATA/IC Software version 17.0 (Stata Corp., College Station, TX, USA) was used for all statistical analyses.

### Ethics approval and consent to participate

The study protocol adhered to the ethical criteria of the 1975 Declaration of Helsinki and was approved by the Institutional Review Board of the Faculty of Medicine at Vajira Hospital (COA 207/2564). The Institutional Review Board of the Faculty of Medicine Vajira Hospital committees waived the requirement for patient informed consent due to the design of the retrospective cohort study and allowed the authors to review their medical records.

## Results

Of the 354 participants receiving NOACs, after excluding those who had been on NOACs for less than 30 days, those who had severe thrombocytopenia, gastrointestinal malignancy, hematologic malignancy, or end-stage renal disease (ESRD), and those with incomplete data, 276 patients were included in this study. Table 1 shows that 185 eligible patients were receiving PPI cotherapy and 91 were receiving NOAC monotherapy. Patients receiving NOACs with PPI cotherapy were older on average than those receiving NOACs alone (*p* = 0.007). There was no statistically significant difference among the 147 females between the groups (*p *= 0.706). The most prevalent indication for the use of NOACs was atrial fibrillation (98.6%). Dabigatran was the most frequently used NOAC (46.7%), followed by rivaroxaban (29.3%), apixaban (20.3%), and edoxaban (3.6%). Hypertension (83.8% vs. 64.8%; *p* < 0.001) and chronic kidney disease (30.3% vs. 9.9%; *p* <0.001) were more prevalent in the cotherapy group than in the NOAC-alone group. Compared to the control group, the cotherapy group had substantially higher rates of concurrent antiplatelet drug use, NSAID use, and long-term steroid use [48.6% vs. 12.1%, 21.1% vs. 3.3%, and 25.4% vs. 7.7%, respectively; all *p* < 0.001]. The HAS-BLED score was significantly higher in the group at elevated risk for UGIB due to cotherapy (3.02 ± 1.15 vs. 2.2 ± 1.08; *p* < 0.001) (Table 1). The median CCI score of the NOAC monotherapy group indicated moderate comorbidities, while that of the cotherapy group indicated moderate to severe comorbidities (*p* < 0.001). The majority of dual-therapy patients had a daily PPI dose of 20 mg. After adjusting for creatinine clearance, the mean dose of each NOAC was comparable in both groups (dabigatran 220 mg, rivaroxaban 20 mg, apixaban 5 mg, and edoxaban 60 mg).

**Table 1:** Baseline characteristics of the NOAC-treated group.

Patient characteristic	Total, n (%) N = 276	NOACs alone, n (%) N = 91	NOACs with PPIs, n (%) N = 185	*p*
Female	147 (53.3)	47 (51.6)	100 (54.1)	0.706
Age (median, IQR)	76.70 (70.03–82.21)	75.02 (67.04–79.96)	77.80 (70.74–82.58)	0.007
Indication for anticoagulants				0.106
Atrial fibrillation	272 (98.6)	88 (96.7)	184 (99.5)	
Venous thromboembolism	4 (1.4)	3 (3.3)	1 (0.5)	
Comorbidities				
Chronic kidney disease	65 (23.6)	9 (9.9)	56 (30.3)	< 0.001
Cirrhosis	5 (1.8)	NA	5 (2.7)	0.113
Ischemic heart disease	2 (0.7)	1 (1.1)	1 (0.5)	NA
Heart failure	17 (6.2)	6 (6.6)	11 (5.9)	0.272
Diabetes mellitus	115 (41.7)	34 (37.4)	81(43.8)	0.309
Hypertension	214 (77.5)	59 (64.8)	155 (83.8)	< 0.001
NOACS				0.009
Rivaroxaban	81 (29.3)	38 (41.8)	43 (23.2)	
Apixaban	56 (20.3)	16 (17.6)	40 (21.6)	
Dabigatran etexilate	129 (46.7)	36 (39.6)	93 (50.3)	
Edoxaban	10 (3.6)	1 (1.1)	9 (4.9)	
Concomitant medication				
NSAID use	42 (15.2)	3 (3.3)	39 (21.1)	< 0.001
Antiplatelet use	101 (36.6)	11 (12.1)	90 (48.6)	< 0.001
Steroid use	54 (19.6)	7 (7.7)	47 (25.4)	< 0.001
HAS-BLED score (Mean ± SD)	2.7 ± 1.19	2.2 ± 1.08	3.02 ± 1.15	< 0.001
Charlson Comorbidity Index score (median, IQR)	4 (3–5)	3 (3–4)	4 (3–6)	< 0.001

Chi-square test or Fisher's exact test for categorical data and Mann‒Whitney U test for continuous data.

*NOACs* nonvitamin K antagonist oral anticoagulants, *NA* not available, *NSAIDs* nonsteroidal anti-inflammatory drugs, *PPI* proton pump inhibitor.

During a mean follow-up period of 29.5 months, 5.1% of the patients experienced UGIB episodes. The incidence of UGIB was higher in patients taking NOACs without PPIs (IR 2.7 [1.26–5.60]/1000 person-months) than in patients taking NOACs with PPIs (IR 1.3 [0.61–2.67]/1000 person-months) (Table 2). After adjustment for sex, age, comorbidities, the concomitant use of antiplatelets, NSAIDs, and steroids and the HAD-BLED score, patients receiving NOACs with PPIs had a lower risk of UGIB than patients receiving NOAC monotherapy (RR 0.208, 95% CI 0.061–0.706; *p* = 0.012). Females were more likely to experience UGIB than males (RR 5.043; 95% CI 1.096–23.20; *p* = 0.038). After adjusting for confounding variables, as shown in Table 3, a one-point increase in the HAS-BLED score was associated with a 2.024-fold increase in the risk of UGIB (RR 2.024; 95% CI 1.095–3.743; *p* = 0.024).

**Table 2:** Occurrence of upper gastrointestinal hemorrhage in NOAC-treated patients classified by PPI use.

	N (%)	Follow-up time (month)	Event N (%)	IR* (95% CI)
Total	276	29.49 ± 25.2	14 (5.1)	–
NOACs	91 (33)	29.02 ± 26.7	7 (7.7)	2.7 (1.26–5.60)
NOACs with PPIs	185 (67)	29.72 ± 24.4	7 (3.8)	1.3 (0.61–2.67)

*NOACs* nonvitamin K antagonist oral anticoagulants, *PPI* proton pump inhibitor.

*IR = Incidence per 1000 person-months.

**Table 3:** Univariate and multivariate analyses of factors associated with UGIB

Factor	No UGIB N (%)	UGIB N (%)	Crude RR (95% CI)	*p*	Adjusted RR* (95% CI)	*p*
PPI cotherapy						
NOACs	84 (92.3)	7 (7.7)	1	-	1	-
NOACs with PPIs	178 (96.2)	7 (3.8)	0.492 (0.173–1.402)	0.184	0.208 (0.061–0.706)	0.012
Sex						
Male	127 (98.4)	2 (1.6)	1		1	-
Female	135 (91.8)	12 (8.2)	5.265 (1.178–23.526)	0.030	5.043 (1.096–23.20)	0.038
Age (median, IQR)	76.94 (69.13–82.22)	73.47 (70.74–80.40)	1.014 (0.960–1.071)	0.621	1.002 (0.928–1.082)	0.959
Comorbidities						
Yes	220 (94.4)	13 (5.6)	2.259 (0.708–7.201)	0.168	0.778 (0.079–7.677)	0.829
No	42 (97.7)	1 (2.3)	1	-	1	-
Concomitant medication use						
Yes	133 (93.0)	10 (7.0)	2.399 (0.314–18.340)	0.154	1.598 (0.342–7.463)	0.551
No	129 (97.0)	4 (3.0)	1	-	1	-
HAS-BLED score (median, IQR)	3 (2–3)	4 (2.75–4.25)	1.853 (1.196–2.869)	0.006	2.024 (1.095–3.743)	0.024
Charlson Comorbidity Index score (median, IQR)	4 (3–5)	4.5 (4–6)	1.311 (0.972–1.770)	0.076	1.025 (0.695–1.512)	0.899

*CI* confidence interval, *RR* risk ratio, *NOACs* nonvitamin K antagonist oral anticoagulants, *NSAIDs* nonsteroidal anti-inflammatory drugs, *PPI* proton pump inhibitor, *UGIB* upper gastrointestinal bleeding.

*Adjusted for sex, age, comorbidities, concomitant medication use and HAS-BLED and Charlson Comorbidity Index scores.

Over fifty percent of UGIB patients underwent upper endoscopy [NOACs (3), NOACs with PPIs (5)]. In both groups, seven out of eight individuals experienced esophagitis and gastritis, with the exception of one individual diagnosed with a peptic ulcer. In this study, there were no high-risk endoscopic stigmata. The length of hospitalization was comparable between the groups (6 days for patients receiving NOACs alone vs. 9 days for those receiving NOACs plus PPIs; *p* = 0.7). In terms of thromboembolic events, cerebrovascular ischemia was more prevalent in the combination therapy group, but the difference was not statistically significant (3.24% vs. 2.19%; *p* = 0.24).

## Discussion

According to earlier reports, the incidence of UGIB events in NOAC-treated patients was between 1.19 and 2.84%^15–17^. Most studies assess the risk of UGIB within 1 year of follow-up^17^. The incidence of UGIB in our investigation was slightly higher than that in a previous NOAC-treated report with a comparable follow-up period^18^. Prior research demonstrated that some NOACs increased the risk of UGIB in elderly individuals, particularly dabigatran, which is associated with an increased risk of hemorrhage in the elderly population^4,5^. According to our data, the average age of participants was greater than 75 years old, which may be a factor in the increasing prevalence of UGIB. In addition to age, the 1-year risk of significant bleeding in anticoagulant-treated atrial fibrillation patients is determined by a simple HAS-BLED score. A score of 3 indicates "high risk"; this score can predict the risk of bleeding and is simple to apply. Using this index to assess our NOAC-treated patients, we determined that more than fifty percent fell into high-risk categories. The mean HAS-BLED score and percentage of participants at high risk for hemorrhage in the PPI cotherapy group were greater than those in the group receiving NOACs alone.

Despite previous studies indicating a higher risk of UGIB in NOAC-treated patients with concomitant diseases, particularly those with renal impairment^7,19^, it was suggested that dabigatran not be administered if the glomerular filtration rate (GFR) is less than 25. Even though the high prevalence of chronic kidney disease in our cohort may be attributable to the fact that over a third of our patients were diabetic and geriatric, average GFRs between 40 and 60 mL/min may not necessitate a dose adjustment.

Numerous studies have demonstrated that apixaban is the safest medication, with the lowest frequency of UGIB events, when compared to OACs or other NOACs in terms of NOAC type and UGIB risk^17,20–26^. Dabigatran was the first NOAC authorized for use in thromboembolism prophylaxis following the introduction of NOACs; thus, approximately half of the patients in our study were administered dabigatran etexilate. The results on dabigatran supported and contradicted the reduced prevalence of UGIB episodes when compared to OACs. Some studies found that dabigatran was associated with a lower risk of major bleeding^20,22–24^, however, other studies found that dabigatran were shown significantly increase the risk of bleeding when compared to OACs^17,21,27–29^, particularly in the elderly population and at a dose of 300 mg per day^28,30^. The cytotoxic effects of dabigatran etexilate are mediated by the production of reactive oxygen species associated with mitochondria, which may cause upper gastrointestinal tract mucosal injury^31^. To reduce the risk of hemorrhage, our physicians prescribed 110 mg of dabigatran once or twice daily, based on evaluations^32^. Consistent with previous reports^33–35^, the preponderance of our endoscopic findings is indicative of esophagitis. Even though dabigatran was associated with the highest proportion and trend of UGIB events among NOAC users, the difference in cause‒effect occurrence was not statistically significant.

According to meta-analyses^36^, the protective effect of PPIs against the occurrence of GIB events was greater in patients taking dabigatran than in those taking XA inhibitors. Even though more than a third of our subjects reported concurrent antiplatelet or aspirin use, the influence of coulcerogenic medication did not compromise the preventative efficacy of the PPI in NOAC-PPI cotherapy. As evidenced by prior research, the combination of NOACs with either aspirin or antiplatelets does not increase the risk of UGIB^1^. In addition, underlying comorbidities or concomitant medications predicted to increase ulcer risk at baseline were less prevalent in our patients receiving NOAC monotherapy, and these factors had no influence on UGIB risk. However, additional studies of NOAC-treated patients are necessary to corroborate this conclusion. None of our UGIB patients presented with high-risk bleeding stigmata, and all bleeding was treated conservatively. Consistent with previous findings, NOACs produce less severe GIB^11^. Despite the possibility that the acid-lowering effect of PPIs could reduce the efficacy of dabigatran relative to NOACs alone, there was no significant increase in the risk of thromboembolic events in the combination therapy group.

Our research was limited by a number of factors. First, the NOAC reimbursement policy was not wholly open during the study periods, so we recruited fewer NOACS-treated patients than anticipated. Second, the limited number of censored events may have restricted the influence of confounding variables in the multivariable analysis. Third, because the tablets for medical consumption were not counted, the precision of compliance may be diminished.

Because GIB outcomes were less common in patients treated with NOACs, the retrospective study required a larger sample size to explain the efficacy of PPIs in preventing UGIB in patients treated with NOACs. In the future, a randomized controlled trial of PPI-NOAC cotherapy and NOAC therapy alone will be of interest for minimizing the selection and information bias that naturally occurs in studies with retrospective cohort designs.

## Conclusions

Patients taking both NOACs and PPIs had a reduced risk of upper gastrointestinal bleeding compared to those taking NOACs alone. Patients who had higher HAS-BLED scores and women were more likely to experience UGIB while taking NOACs.

### Supplementary Information

Supplementary Information.

## Data Availability

The data used in this work are available upon reasonable request from the corresponding author.
