# Supplementary material for: Nonvitamin K oral anticoagulants with proton pump inhibitor cotherapy ameliorated the risk of upper gastrointestinal bleeding
Source: Sci Rep. 2023 Oct 13;13:17329. doi: 10.1038/s41598-023-44494-9 (PMC10575972; doi:10.1038/s41598-023-44494-9)
Supplement: Supplementary file 1 — Supplementary Information. [file 41598_2023_44494_MOESM1_ESM.docx]

**Table Supplement 1 Definitions of comorbidities and outcomes by ICD-10**

| **Variable** | **ICD-10 codes** |
| --- | --- |
| ***Co-morbidity*** | |
| Chronic Renal failure | N18.1 – N18.5, N18.9, N19 |
| Hypertension | I10-I13, I15 |
| Cardiovascular disease  - Ischemic heart disease  - Heart failure | I20 – I25  I50, I42.0, I11.0, I13.0, I13.2 |
| Diabetes Mellitus | E10, E100-E109, E11, E110-E119, E12, E120-E129  E13, E130-E139, E14, E140-E149 |
| ***Endpoint*** | |
| **Upper gastrointestinal bleeding** | |
| Hematemesis | K92.0 |
| Melena | K92.1 |
| Upper gastrointestinal bleeding | K92.2 |
| Unspecified gastrointestinal bleeding | K92.2 |
